# Associations Between Pain Intensity and Inflammatory Profile in Women with Android and Gynoid Obesity Diagnosed with Chronic Pain: An Observational Study

**DOI:** 10.3390/jcm14124170

**Published:** 2025-06-12

**Authors:** Cecília Cristina Cota, Stefani Miranda-Castro, Antônio Felipe Souza-Gomes, Luciano Bernardes Leite, Pedro Forte, António M. Monteiro, William Valadares Campos Pereira, Samara Silva de Moura, Albená Nunes-Silva

**Affiliations:** 1 Inflammation and Exercise Immunology Laboratory (LABIIEX), Department of Physical Education and Sports, School of Physical Education, Federal University of Ouro Preto, Ouro Preto 35400-000, MG, Brazilstefani.castro@aluno.ufop.edu.br (S.M.-C.); antonio.fsg@aluno.ufop.edu.br (A.F.S.-G.); william.valadares@yahoo.com.br (W.V.C.P.); 2 Department of Physical Education, Federal University of Viçosa, Viçosa 36570-900, MG, Brazil; luciano.leite@ufv.br; 3 Department of Sports, Instituto Politécnico de Bragança, 5300-253 Bragança, Portugal; mmonteiro@ipb.pt; 4 Department of Sports, Higher Institute of Educational Sciences of the Douro, 4560-547 Penafiel, Portugal; 5 CI-ISCE, Instituto Superior de Ciências Educativas do Douro (ISCE Douro), 4560-547 Penafiel, Portugal; 6 Research Center for Active Living and Wellbeing (LiveWell), Instituto Politécnico de Bragança, 5300-253 Bragança, Portugal; 7 Epidemiology Laboratory, Research and Study Group on Nutrition and Public Health (GPENSC), Department of Nutrition, Federal University of Ouro Preto, Ouro Preto 35400-000, MG, Brazil; samara_silva09@hotmail.com

**Keywords:** android, chronic pain, C-reactive protein, gynoid, inflammation, interleukin-6

## Abstract

**Background:** There are different types of obesity, and the metabolic conditions associated with these phenotypes are also heterogeneous. Overweight and obesity are not only associated with pain but are also identified as risk factors for the development of pain. Objective: This study aimed to compare the levels of inflammatory biomarkers, counting of immune cells, and chronic pain between android and gynoid female patients with obesity. **Method:** Thirty (n = 30) women took part in this study (18 androids, age: 50.61 ± 9.41 and 12 gynoids, age: 50.67 ± 9.45). The participants underwent anamnesis, Visual Numeric Scale (VNS), dual-energy X-ray absorptiometry, and blood sampling for the analysis of leukocytes, C-reactive protein (CRP), and interleukin (IL)-6. **Results:** The number of total leukocytes in the blood was not different when comparing the android group (6045 µL) with the gynoid group (5230 µL). No differences were observed for neutrophils (3440 µL in android and 3017 µL in gynoid), lymphocytes (2208 µL in android and 2115 µL in gynoid), for monocytes (429.7 µL in android and 392.8 µL in gynoid), and basophils (17.27 µL in android and 15.41 µL in gynoid). However, there was a significant difference between the number of eosinophils when comparing the android group (137.6 µL) with the gynoid group (204.9 µL), *p* = 0.04. Although both groups presented CRP values above 0.3 mg/dL—indicative of low-grade inflammation—no statistically significant difference was observed. Similarly, no difference was found in pain intensity between groups, as measured by the Visual Numeric Scale (VNS). **Conclusions:** Although most inflammatory and pain markers did not differ between groups, the higher eosinophil count in the gynoid phenotype suggests immunological distinctions between obesity types. These findings underline the importance of considering body fat distribution in clinical assessments of inflammation and chronic pain in women with obesity.

## 1. Introduction

Obesity is a global health problem and a risk factor for the development of chronic non-communicable diseases, such as type 2 diabetes mellitus (T2DM), cardiovascular diseases, some types of cancer, autoimmune diseases, mood disorders, dementia, and musculoskeletal disorders [1]. Overweight (body mass index [BMI] > 25 kg/m^2^) and obesity (BMI > 30 kg/m^2^) are the main factors contributing to the global burden of morbidity [2], and their prevalence has been increasing worldwide, possibly due to behavioral changes in recent decades, such as poor diet and a sedentary lifestyle [3]. Worldwide data from 2016 showed that 39% of adults were overweight and 13% were obese, with BMI showing an increasing trend between 1975 and 2016 [4]. Initially, these conditions were only considered a public health problem in high-income countries, but since the 2000s, overweight and obesity have been growing at a faster rate in low- and middle-income countries [5]. The health costs for a single individual with obesity are 1.5 to 1.8 times higher than for an individual without obesity, and in addition to the direct economic costs, there are also indirect costs related to absenteeism, loss of productivity, and premature mortality [6].

Adipose tissue is distributed throughout the body and plays an important role in physiological regulation such as systemic metabolism, energy balance, and immune responses [7]. In obesity, when the adipose tissue is in excess owing to an increase in the number of cells (hyperplasia) or their volume (hypertrophy), adipocytes undergo structural and functional remodeling, which ultimately results in impaired angiogenesis, development of hypoxia and fibrosis, and changes in the secretory profile and inflammatory and immune responses [8]. An increase in adipocyte size results in important metabolic changes in adipose tissue, characterized by higher levels of inflammation [9]. Circulating levels of inflammatory mediators, such as C-reactive protein (CRP) and its inducer interleukin (IL)-6, are increased in individuals with obesity compared to those without obesity [10]. In addition, M1-type macrophages, which produce proinflammatory cytokines (including IL-6), are found in greater quantities in excessively enlarged adipose tissue [11]. This change in the phenotype of immune system cells suggests a hyperactive behavior of adipose tissue as an endocrine organ, which could promote chronic systemic inflammation because the substances excreted have local and systemic actions [12,13]. CRP and IL-6 are considered inflammatory biomarkers to assess the presence and severity of low-grade inflammation in obesity [14,15].

IL-6 is a low-molecular-weight protein (20–30 kDa) that acts locally and systemically to generate a variety of physiological responses related to endocrine and metabolic functions [16]. Because of these characteristics, IL-6 plays a central role in the relationship between obesity, inflammation, and coronary heart disease and chronically contributes to the maintenance of insulin resistance [17,18]. In addition to adipocytes, various cell types can secrete IL-6, such as macrophages, lymphocytes, pancreatic endothelial and beta cells, hepatocytes, muscle fibers, and astrocytes [19,20]. Thus, adipose tissue represents an important source of IL-6 for systemic circulation, and there may be a direct correlation between IL-6 production and fat mass.

One of the best-known and most studied functions of IL-6 is the stimulation of CRP production by hepatocytes. CRP is an acute-phase protein with a molecular weight of approximately 115 kDa, produced in the liver and secreted into the bloodstream during an inflammatory episode, largely in response to IL-6 signaling [21]. Phase proteins are those whose serum concentrations increase or decrease by at least 25% during the inflammatory state. However, despite their names, they can undergo changes during chronic inflammation [22].

In general, mild inflammation and viral infections lead to elevations in the 10–40 mg/L range, whereas more severe inflammation and bacterial infections lead to serum concentrations of 40–200 mg/L [22]. In addition, systemic inflammatory biomarkers may favor persistent chronic pain as they appear to stimulate neural transmission through the dorsal root ganglion and reduce pain thresholds [23].

Excess adipose tissue, as observed in individuals with overweight and obesity, is a state in which the immune cells of this tissue behave irregularly [8,24], reflecting a greater propensity for pain, malaise, and depression in overweight individuals [23,25].

Overweight and obesity are not only associated with pain but are also identified as risk factors for the development of pain [26,27]; overweight people report approximately 20% more pain than normal-weight people, and for individuals with obesity, this percentage reaches 68% [28]. The biomechanical load, referring to increased stress on joints and soft tissues due to excess body weight, induced by obesity is the most direct link between obesity and chronic pain [29]. In fact, given the multifactorial nature of musculoskeletal disease, obesity consistently emerges as a key and potentially modifiable risk factor in the onset and progression of musculoskeletal conditions of the hip, knee, ankle, foot, and shoulder [30]. In this sense, studies have shown an association between obesity and joint pain in non-weight-bearing body segments. Furthermore, obesity shape markedly influences spine biomechanics [31], and it is a risk factor for osteoarthritis of the hand and wrist [32].

Understanding the possible associations among body fat, inflammation, and chronic pain can improve treatment strategies for populations with obesity. This study aimed to compare the circulating levels of inflammatory biomarkers (CRP and IL-6) and pain levels in patients classified as android or gynoid with a clinical diagnosis of chronic joint pain, specifically in the shoulder, spine, and/or knee. We hypothesized that the android group would present higher levels of inflammatory biomarkers and pain intensity due to the greater metabolic activity of visceral fat.

## 2. Materials and Methods

### 2.1. Study Design and Participants

**Figure 1:**
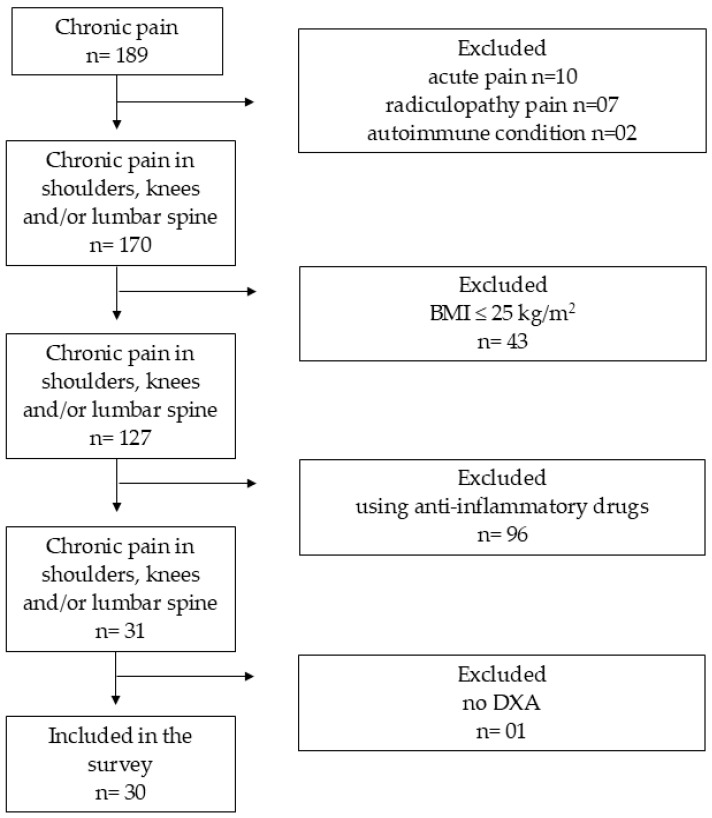
Flowchart for recruiting participants.

This was an observational, analytical, and cross-sectional study with two groups (android or gynoid). Participants were recruited using convenience sampling from the physical rehabilitation sector of a health unit in Mariana, MG, between 2022 and 2023 (Figure 1). The sample consisted of patients who were on a waiting list for physiotherapy care and referred for clinical diagnoses including chronic joint pain in the shoulder, knee, and spine. Those who met the inclusion criteria were invited to undergo a physiotherapy assessment and participate in the study.

The inclusion criteria included the following: being a woman aged between 30 and 65 years; having a clinical diagnosis of chronic joint pain in the shoulder, spine, and/or knee; pain intensity ≥ 4 on the Visual Numeric Scale (VNS); and being classified as overweight or obese based on BMI. Exclusion criteria included the following: having received physical treatment for pain in the last 15 days; use of anti-inflammatory medication within the previous 48 h; diagnosis of unresolved systemic or musculoskeletal inflammatory conditions (e.g., radiculopathies, sprains); autoimmune diseases, coagulopathies, infections, or neoplastic diseases; pregnancy; disinterest in participation; and symptoms suggestive of COVID-19.

This study was approved by the Research Ethics Committee of the Federal University of Ouro Preto (UFOP/MG) under protocol number CAAE 59563422.7.0000.5150, opinion number 5.650.847 (19 September 2022). All participants signed an informed consent form after receiving detailed information about the study’s procedures, risks, and benefits. Refusal to participate did not affect their clinical care or relationship with the researchers.

### 2.2. Procedures

The study procedures, represented in the flowchart (Figure 1), were explained to all participants through the ICF. Upon agreement, they signed the informed consent form. On the same day, anamnesis was conducted, the International Physical Activity Questionnaire (IPAQ) was administered, and the VNS was presented to allow the participant to quantify the intensity of her pain. The dates for blood sample collection and dual-energy X-ray absorptiometry (DXA) tests were scheduled and performed at the Central Laboratory. To classify the participants in the Android and Gynoid phenotype groups, data acquired in the dual-energy X-ray absorptiometry (DXA—Lunar DXA Densitometer—Prodigy Primo from General Electric Healthcare ^TM^) exam were used. The criteria used for this classification was the location of greatest fat concentration in the body of the participants. In this sense, 18 participants were classified as an Android phenotype, as they showed more fat accumulation in the body trunk region (46.22% ± 4.66%) than in the legs and hips region (40.84% ± 5.86%), and another 12 participants were classified as a Gynoid phenotype, as they showed more fat accumulation in the hips and legs region (51.81% ± 5.66%) than in the body trunk region (48.41% ± 6.41%).

### 2.3. Evaluation Parameters and Instruments

#### 2.3.1. International Physical Activity Questionnaire (IPAQ)

The International Physical Activity Questionnaire (IPAQ), proposed by the World Health Organization (WHO) in 1998, is an international instrument for determining the level of physical activity and has been validated in Brazil [33]. The questionnaire consists of eight questions referring to the physical activities performed by the individual on a weekly basis, and of the eight questions, two are related to sedentary behavior. Depending on the frequency of physical activity, duration, and intensity, the individual’s level of physical activity is classified as “Very Active”, “Active”, “Irregularly Active A”, “Irregularly Active B” or “Sedentary.”

#### 2.3.2. Visual Numeric Scale (VNS)

The VNS is a numerical scale (from 0 to 10), where 0 indicates “no pain” and 10 indicates the “worst pain imaginable,” representing a simple and common measure of pain severity [34,35,36]. The participants were instructed to choose a single number from a scale that best indicated their level of pain. One-dimensional pain scales such as the VNS are among the most reliable and valid measurement tools for self-reporting pain intensity [37].

#### 2.3.3. Body Composition Assessment

To assess body composition, whole-body densitometry was carried out using the DXA technique with the General Electric Healthcare Lunar DXA—Prodigy Primo densitometer, considered an accurate test and classified as the “gold standard” for assessing body composition [38]. The test was performed at the CEMEDI Clinic in Mariana, Minas Gerais.

#### 2.3.4. Blood Collection

The participants prepared themselves by following two guidelines prior to blood collection: (1) fasting for 8 h and (2) avoiding the use of anti-inflammatory medication for at least 48 h. Venipuncture was performed on the antecubital fossa in a prepared environment by a qualified professional with experience and appropriate materials, respecting biosafety standards.

#### 2.3.5. Biochemical Analysis

The blood samples were collected in 4 mL tubes of peripheral venous blood for blood count analysis, and in tubes containing EDTA and 4 mL tubes with separating gel for interleukin and CRP analysis; for the latter two, the samples were centrifuged at 3000 revolutions per min (RPM) for 10 min at a temperature of 4 °C, acceleration of 5 RPM and deceleration of 5 RPM. The plasma samples were then pipetted into 1.5 mL Eppendorf tubes and stored in a −80 °C freezer for future cytokine analysis using the electrochemiluminescence method. The inflammatory biomarker was measured using ELISA for cytokine bead array (CBA) (Becton Dickinson and Company®, Franklin Lakes, NJ, USA), used to quantify IL-6, using flow cytometry in the Central Laboratory of the Mariana City Hall Polyclinic.

### 2.4. Statistical Analysis

For the statistical analysis, we used GraphPad Prism 9 software. Initially, we performed the Shapiro–Wilk test to verify the normality of the data. We then applied the *t*-test for data with a parametric distribution and the Mann–Whitney test for non-parametric data. To explore correlations, we used Spearman’s coefficient for non-parametric data and Pearson’s correlation for parametric data, with the significance level set at *p* ≤ 0.05. The results for parametric data were presented as mean and standard deviation, whereas for non-parametric data, median and standard error were used as descriptive measures.

## 3. Results

In total, 30 women with a clinical diagnosis of chronic pain, classified as overweight or obese according to their BMI, and who quantified pain greater than or equal to 4 on the VNS were included in this study. The participants were categorized into two groups according to their fat distribution pattern: android (*n* = 18; mean age: 50.61 years) and gynoid (*n* = 12; mean age: 50.67 years). The mean height was 157.88 cm in the android group and 156 cm in the gynoid group, and the mean total body mass was 81.53 kg in the android group and 83.10 kg in the gynoid group. The other characteristics of the study participants are presented in Table 1.

**Figure 2:**
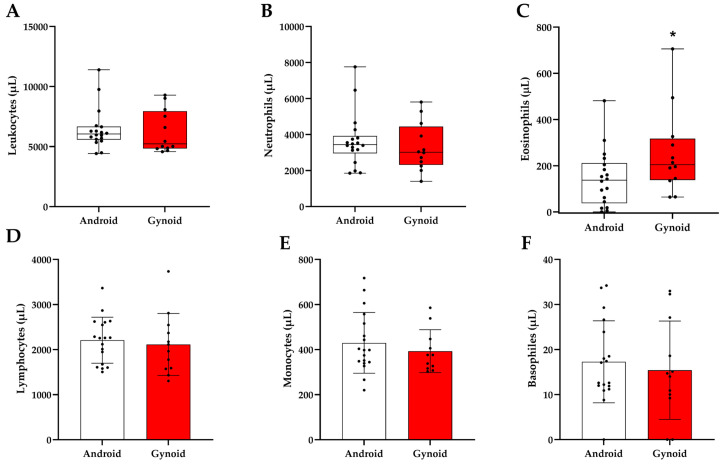
Total leukocyte count and subpopulations according to fat distribution (*n* = 30; *n* = 18 android and *n* = 12 gynoid). (**A**) Leukocytes. (**B**) Neutrophils. (**C**) Eosinophils. (**D**) Lymphocytes. (**E**) Monocytes. (**F**) Basophils. Data are expressed as medians for panels (**A**–**C**) (Mann–Whitney test), and as means ± standard deviation for panels (**D**–**F**) (Student’s *t*-test). * *p* < 0.05.

Figure 2 shows the total circulating leukocyte counts and subpopulations of neutrophils, lymphocytes, monocytes, eosinophils, and basophils in the participants’ blood samples. The number of total leukocytes in the blood was not statistically different when comparing the android group (6045 µL) with the gynoid group (5230 µL) with *p* = 0.48 (Figure 2A). No differences were observed for neutrophils (3440 µL in android and 3017 µL in gynoid, with *p* = 0.41) (Figure 2B), nor for lymphocytes (2208 µL in android and 2115 µL in gynoid with *p* = 0.67) (Figure 2D), for monocytes (429.7 µL in android and 392.8 µL in gynoid, with *p* = 0.43) (Figure 2E), and basophils (17.27 µL in android and 15.41 µL in gynoid, with *p* = 0.61) (Figure 2F). However, there was a significant difference between the number of eosinophils when comparing the android group (137.6 µL) with the gynoid group (204.9 µL), *p* = 0.04 (Figure 2C).

**Figure 3:**
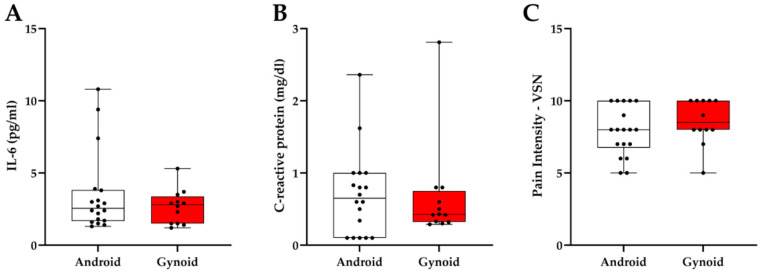
Inflammatory markers and pain intensity according to fat distribution (*n* = 30; *n* = 18 android and *n* = 12 gynoid). (**A**) Interleukin-6 (IL-6). (**B**) C-reactive protein (CRP). (**C**) Pain intensity assessed by Visual Numeric Scale (VNS). Data are expressed as medians (Mann–Whitney test for all panels).

Figure 3 shows that serum concentrations of IL-6 (Figure 3A) and CRP levels (Figure 3B) were not significantly different between the android and gynoid groups. Although both groups presented CRP values above 0.3 mg/dL—indicative of low-grade inflammation—no statistically significant difference was observed. Similarly, no difference was found in pain intensity between groups, as measured by the Visual Numeric Scale (VNS) (Figure 3C).

**Figure 4:**
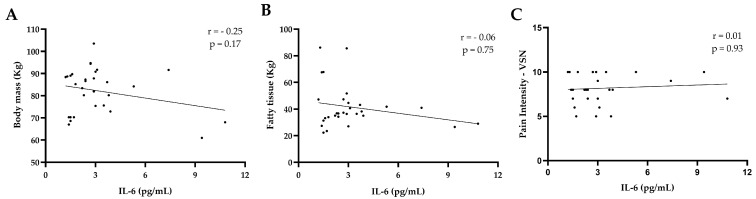
Correlation between IL-6 levels and clinical parameters (*n* = 30). (**A**) Body mass. (**B**) Fatty tissue. (**C**) Pain intensity assessed by Visual Numeric Scale (VNS). Pearson’s correlation was used for panel (**A**), and Spearman’s correlation for panels (**B**,**C**). Lines represent linear trends.

Figure 4 shows the correlation between interleukin-6 (IL-6) and body mass, fat tissue, and pain intensity. No association was found between IL-6 and body mass (Figure 4A) (*p* < 0.17), IL-6 and fat tissue (Figure 4B) (*p* < 0.75), and IL-6 and pain intensity (Figure 4C) (*p* < 0.93).

**Figure 5:**
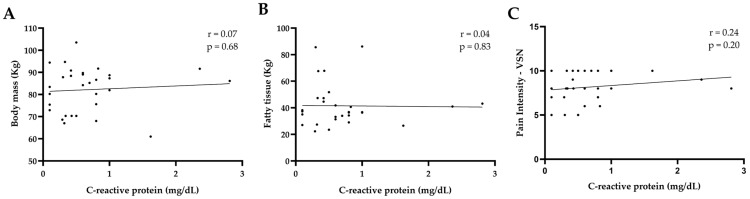
Correlation between C-reactive protein (CRP) levels and clinical parameters (*n* = 30). (**A**) Body mass. (**B**) Fatty tissue. (**C**) Pain intensity assessed by Visual Numeric Scale (VNS). Pearson’s correlation was used for panel (**A**), and Spearman’s correlation for panels (**B**,**C**). Lines represent linear trends.

Figure 5 shows the correlation between CRP and body mass, fat tissue, and pain intensity. No correlation was found between CRP and body mass (Figure 5A) (*p* < 0.68), CRP and fat tissue (Figure 5B) (*p* < 0.83), and CRP and pain intensity (Figure 5C) (*p* < 0.20).

**Figure 6:**
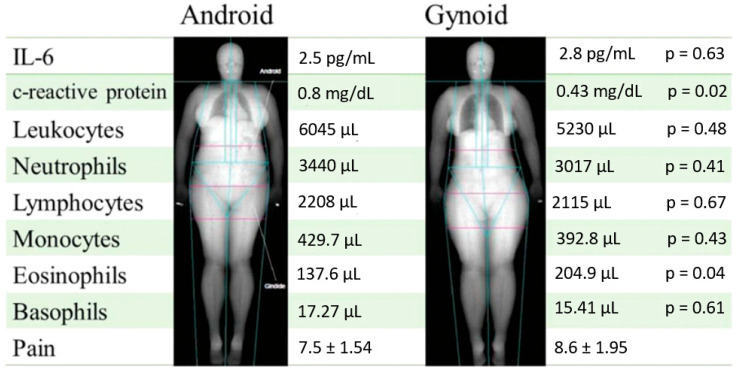
Summary of findings according to fat distribution (*n* = 30; *n* = 18 android and *n* = 12 gynoid). Mean values are presented for each variable by group. Data include inflammatory markers (IL-6 and CRP), leukocyte subpopulations, and pain intensity. *p*-values refer to comparisons between android and gynoid groups.

Figure 6 summarizes the main findings by fat distribution pattern. Compared to the android group, the gynoid group showed lower C-reactive protein levels and higher eosinophil counts. No other significant differences were observed between the groups.

## 4. Discussion

This study aimed to compare the circulating levels of inflammatory biomarkers (CRP and IL-6) and pain in patients classified as androids or gynoids with a clinical diagnosis of chronic joint pain. A state of low-grade inflammation was found in both androids and gynoids, as confirmed by serum CRP, with a reference value of > 0.3 mg/dL according to the American Heart Association (AHA). In addition, the results showed a statistically significant difference between the two groups, with higher CRP levels in the android group. There was no statistically significant difference in pain intensity measured using the VNS between the groups. These findings partially confirm our hypothesis: While the higher CRP levels observed in the android group were expected due to the greater metabolic activity of visceral fat, the absence of differences in pain intensity may be attributed to the participants’ level of physical activity. As shown by the IPAQ scores, most participants were not sedentary, and regular physical activity is known to attenuate both systemic inflammation and pain perception.

Another important analysis was the difference in pain intensity between android and gynoid phenotypes. Although there was no difference between the android and gynoid groups, high pain intensity was observed in both groups. These data corroborate the findings of Meleger et al. [39], who pointed out that obesity, without distinguishing between phenotypes, is often associated with chronic pain.

Through the answers given in the IPAQ questionnaire, it was possible to observe that more than 70% of the participants in this study were not sedentary. Knowing the level of physical activity of participants is important because physical exercise has direct and indirect benefits for most individuals with chronic pain [40]. In addition, physical exercise can reverse some obesity-related pathologies by modulating the crosstalk between adipose tissue and various physiological systems [41]. Thus, based on the results, it is possible to see that there was an effort on the part of the participants to lead a physically active lifestyle.

A previous study reported that obesity is associated with a chronic low-grade inflammatory state, characterized by increased circulating immune cells, including neutrophils and lymphocytes, as part of an enhanced immune activation profile [42]. Additionally, one clinical study found a positive correlation between lymphocyte count and BMI, with overweight individuals presenting higher lymphocyte levels compared to normal-weight individuals [43].

Among the results found in the blood count, the eosinophil count stands out. Despite being described in the literature as being poorly expressed in obesity [44], increased values were observed in the gynoid group when compared to the android group. These findings may suggest that subcutaneous fat plays an important role in immunometabolic homeostasis in obesity since it is well established that eosinophils are the main secretors of the anti-inflammatory interleukin IL-4 [45,46]. This eosinophilic response deserves more attention because a recent study [46] has shown that eosinophils, although a subpopulation in small numbers compared to other cells in the bloodstream, appear to be protagonists in some immune responses associated with obesity. This is a point that will possibly bring developments to our research group since other projects will continue the study of the immune response in people with obesity, and these new findings seem to show a relevant role for eosinophils in the immune–metabolic balance of people with obesity.

The results of this study showed no IL-6 levels above the reference values presented in the literature and no difference between the android and gynoid groups. These results were unexpected, as the literature has described that low-grade inflammation in individuals with overweight and obesity often results in increased levels of IL-6, TNF, and IFN, which are related to a pro-inflammatory profile [8]. However, it is possible that our sample population is classified as the so-called “metabolically healthy obesity” [47], which are individuals with obesity but without an increased risk of metabolic complications. This may also explain the absence of the expected correlation between IL-6 and body mass and fat tissue, as observed by Kunz et al. [48]. In addition, knowing that physical exercise has an anti-inflammatory effect [49], these results can be partly explained by the chronic benefits of regular physical exercise, as described by the analysis of the IPAQ answered by the participants in this study.

In the same vein, it has already been described that high pain intensities have been found in individuals with low-grade chronic inflammation, using CRP as a marker [25], which corroborates the findings of the present study. However, in our study, no correlations were found between pain intensity and CRP or IL-6 levels in either the android or gynoid group. It is believed that the fact that the sample was not made up of sedentary individuals may be involved in this balance, because, as is well known, physical exercise is a recognized regulator of inflammation due to the release of myokines (exerkines) that are able to inhibit pro-inflammatory molecules that are directed to the bloodstream and to receptors in various organs [50]. These mechanisms can help suppress the inflammatory action of cytokines released owing to immunometabolic dysregulation promoted by adipose tissue in individuals with obesity, thus rescuing the anti- and pro-inflammatory balance. Cohen et al. [51] reported that pain presents as a physical symptom as the outcome of a dynamic interaction between biological, psychological, and social factors.

Similar to those of Graßmann et al. [52] and Kawai et al. [53], our findings showed low-grade inflammation in individuals with excess adiposity by analyzing the plasma concentration of CRP as a biomarker of inflammation. While other studies have considered BMI in a comprehensive way, we analyzed CRP in different phenotypes of obesity, with precision on actual excess adiposity and the location of its accumulation. We found CRP values indicative of low-grade inflammation in both phenotypes, with a statistically significant difference and an increase in the androids. Our findings increase the robustness of a previous study that established, through other evidence, that the inflammatory reaction is more complex and intense in visceral adipose tissue than in subcutaneous tissue; they have also demonstrated a stronger association between cardiometabolic disorders and waist circumference than with BMI [54]. Corroborating Piening et al. [55], this study found no correlation between CRP and body mass or fat tissue. What may support the high intensities of pain in non-sedentary individuals with obesity, regardless of the location of the accumulation of adipose tissue, but only with altered CRP levels predicting low-grade inflammation and in the absence of correlations, may refer to the fact that pain has multiple origins.

### Strengths, Limitations, and Future Directions

One of the strengths of this study is that DXA was used to analyze the body composition and define the phenotype of the participants based on the anatomical location of fat accumulation, making it possible to carefully evaluate the differences between the groups. It was also possible to identify the sample’s level of physical fitness using the IPAQ; knowledge of physical fitness is important in such studies as physical exercise is an important regulator of inflammation in the body. We also assessed the intensity of chronic pain, considering body composition, differences in the location of fat accumulation, and immunological and inflammatory biomarkers. Therefore, the gynoid group may be exposed to a balance of inflammatory levels, indicated by its lower CRP levels, which could be attributed to the secretion of IL-4, a cytokine with anti-inflammatory properties, by eosinophils. However, our study did not analyze the levels of this cytokine. The main findings of this study are summarized in Figure 6. 

Although this study was carefully designed and conducted, it has some limitations. First, the administration of anti-inflammatory drugs to control pain symptoms in the study population limited our sample size, as these drugs interfered with the analysis of serum concentrations of the evaluated cells and molecules. Additionally, the cross-sectional nature of the study prevented us from determining the causality between pain, body composition, and inflammation. Moreover, considering that pain is multifactorial, other factors could have been assessed to investigate the causality of pain inherent only to inflammation.

For future research, it is important to conduct a longitudinal study to monitor possible metabolic and inflammatory changes, considering that this may be a transitory condition, from a state of no inflammation and obesity to a state of inflammation and obesity. In addition, this study could be expanded to analyze circulating anti-inflammatory biomarkers or evaluate pro-inflammatory biomarkers at the tissue level. Evaluating other causes that may influence pain symptoms, in addition to inflammation, is also crucial.

## 5. Conclusions

Our findings revealed that both android and gynoid phenotypes showed higher levels of CRP as an inflammatory marker without any difference between these two groups. In addition, no difference was found in pain intensity between groups. The number of total leukocytes, neutrophils, lymphocytes, monocytes, and basophils was not different between the android and gynoid group. However, there was a significant difference between the number of eosinophils when comparing the android group with the gynoid group.

## Figures and Tables

**Table 1:** Characteristics of the research subjects (*n* = 30).

Variables	Android		Gynoid	
	Mean ± SD	Min–Max	Mean ± SD	Min–Max
Age (years)	50.61 ± 9.41	30–63	50.67 ± 9.45	30–62
Height (cm)	157.88 ± 4.91	146.5–165.5	156.00 ± 4.59	148.5–159
Total body mass (kg)	81.53 ± 9.40	61–94.4	83.10 ± 10.67	75.6–103.5
BMI (kg/m^2^)	33.19 ± 3.85	26.7–43.1	34.70 ± 4.62	34.7–41.7
Fat mass (kg)	38.44 ± 14.07	23.47–86.15	50.41 ± 18.42	36.39–85.56
Fat percentage (%)	42.79 ± 3.04	34.9–49.8	47.95 ± 0.05	47.2–51.7
VSN (0–10)	7.88 ± 1.66	5–10	8.58 ± 1.49	5–10
IPAQ (min/week)	165.83 ± 108.55	0–450	212.50 ± 153.80	0–540

BMI: body mass index; VNS: visual numerical scale; IPAQ—SV (International Physical Activity Questionnaire Adapted—short version). The results for parametric data were presented as mean and standard deviation, whereas for non-parametric data, median and standard error were used as descriptive measures. The level of significance was set at *p* < 0.05.

## Data Availability

The data will be shared on reasonable request to the corresponding author.
